# Intracellular Bacterial Infections: A Challenge for Developing Cellular Mediated Immunity Vaccines for Farmed Fish

**DOI:** 10.3390/microorganisms6020033

**Published:** 2018-04-22

**Authors:** Hetron Mweemba Munang’andu

**Affiliations:** Section of Aquatic Medicine and Nutrition, Department of Basic Sciences and Aquatic Medicine, Faculty of Veterinary Medicine and Biosciences, Norwegian University of Life Sciences, Ullevålsveien 72, P.O. Box 8146, Dep NO-0033, 046 Oslo, Norway; hetroney.mweemba.munangandu@nmbu.no; Tel.: +47-98-86-86-83

**Keywords:** intracellular, *Edwardsiella*, *Piscirikettsia*, *Yersinia*, vaccine, cellular, immunity

## Abstract

Aquaculture is one of the most rapidly expanding farming systems in the world. Its rapid expansion has brought with it several pathogens infecting different fish species. As a result, there has been a corresponding expansion in vaccine development to cope with the increasing number of infectious diseases in aquaculture. The success of vaccine development for bacterial diseases in aquaculture is largely attributed to empirical vaccine designs based on inactivation of whole cell (WCI) bacteria vaccines. However, an upcoming challenge in vaccine design is the increase of intracellular bacterial pathogens that are not responsive to WCI vaccines. Intracellular bacterial vaccines evoke cellular mediated immune (CMI) responses that “kill” and eliminate infected cells, unlike WCI vaccines that induce humoral immune responses whose protective mechanism is neutralization of extracellular replicating pathogens by antibodies. In this synopsis, I provide an overview of the intracellular bacterial pathogens infecting different fish species in aquaculture, outlining their mechanisms of invasion, replication, and survival intracellularly based on existing data. I also bring into perspective the current state of CMI understanding in fish together with its potential application in vaccine development. Further, I highlight the immunological pitfalls that have derailed our ability to produce protective vaccines against intracellular pathogens for finfish. Overall, the synopsis put forth herein advocates for a shift in vaccine design to include CMI-based vaccines against intracellular pathogens currently adversely affecting the aquaculture industry.

## 1. Introduction

Vaccination is an important disease control strategy that has significantly contributed to reduction of outbreaks and antibiotics use in aquaculture. Its first application in teleosts fish, was by Snieszko [1,2] who successfully immunized carp (*Cyprinus carpio*) against *Aeromonas punctata* in 1938. Similarly, Duff [3] successfully vaccinated rainbow trout (*Oncorhynchus mykiss*) against *Aeromonas salmonicida* by injection and oral vaccine delivery in 1942. Since then, vaccine production has progressively expanded into a large commercial pharmaceutical industry alongside the expansion of aquaculture. Although bacterial vaccines have gained considerable success compared to viral vaccines in recent years, growing evidence shows that there are several emerging bacterial diseases in aquaculture not protected by current commercial vaccines. Thus, there is an urgent need to unravel the immunological pitfalls that hinder the success of these vaccines.

Empirical vaccines based on “killed” whole cell bacteria are by far the most widely known to confer protective immunity against bacterial diseases in aquaculture [4,5,6]. However, the type of adaptive immune response induced by vaccination is highly influenced by the site of antigen uptake. Antigens deposited extracellularly primarily evoke humoral immune responses that neutralize pathogens in body fluids while antigens deposited intracellularly evoke both humoral and cellular mediated immune (CMI) responses of which the latter response is specialized in “killing” and eliminating pathogen-infected cells [7]. The overall success of vaccination in aquaculture is largely attributed to whole cell inactivated (WCI) vaccines targeting extracellular pathogens while the route to success of vaccination against intracellular bacterial pathogens remains a challenge. Unlike the extracellular pathogens neutralized by antibody responses, intracellular pathogens evade antibody neutralization by replicating inside host cells. Therefore, the CMI response, which has the capacity to “kill” and eliminate infected cells is more protective against intracellular pathogens than humoral immunity. Hence, as the number of emerging intracellular pathogens continues to increase the need for CMI-based vaccines is bound to become paramount in aquaculture.

This review provides an overview of intracellular bacteria species infecting fish in aquaculture highlighting their mode of invasion, replication, and survival in infected cells. In addition, it brings into perspective an overview of the current understanding of fish CMI and immunological pitfalls limiting the design of protective vaccines against intracellular pathogens. The overall aim is to advocate for a shift in vaccine design from the widely used WCI vaccines that depend on antibody responses for neutralization of extracellular pathogens, to CMI-based vaccines able to kill and eliminate infected cells in order to reduce the prevalence of intracellular bacterial infections currently having adverse impacts on the aquaculture industry.

## 2. Overview of Intracellular Invasion, Survival, and Replication Strategies of Fish Bacteria

The mechanisms underlying entry into, and survival and replication in intracellular niches reveal specialized and common strategies shared by different bacterial species in establishing intracellular infections [8]. Intracellular bacteria localization can be divided into the following: (i) bacteria having cytoplasmic localization in the phagosome and hereby exist in the cytoplasm, such as *Listeria monocytogenes* [9,10]; (ii) intravascular bacteria that localize in nonacidic vacuoles of endosomes, such as *Mycobacterium* spp., which have been shown to inhibit maturation of phagolysosome fusion after being engulfed by phagocytosis [10,11,12]; and (iii) bacteria with intra-lysosomal localization in acidic and hydrolytic compartments that interact with endosomal networks of host cells, e.g., *Yersinia* [10]. Although these strategies are well studied in detail for pathogens of higher vertebrates, growing evidence shows that fish bacterial species belonging to the same genera with mammalian bacterial species could be using similar mechanisms in establishing their intracellular niches [13,14,15].

To survive intracellularly, pathogens require fitness genes to counteract host-killing mechanisms. Among these is the transport secretion system (TSS) used by many bacteria species to deliver toxins intracellularly in eukaryotic cells [16]. Secretion pathways involve 1–8 TSS genes that modulate cellular functions for the benefit of pathogens [17]. These genes encode various protein subsets that are secreted as effector molecules or translocation apparatus that form pores used for the transportation of effector molecules through host cell membranes [18]. Among these is T3SS, which functions as a macromolecular nanomachine found in most Gram-negative bacteria [19]. It has 20 different sub-proteins of which the core functional structure is a complex needle supramolecular structure used to deliver specific substrates in a sequential order across bacterial envelopes into host cells [16,20]. The T3SS translocate virulence proteins via the needle-shaped supramolecular structure into host cells. Table 1 shows a summary of intracellular bacteria infecting fish in aquaculture, while the common TSS genes found in fish intracellular bacteria are shown in Table 2.

Apart from acquisition of fitness genes, intracellular replicating bacteria develop other survival strategies that include (i) modulations of phagosome biogenesis to enable bacteria to resist acidic phagolysosomal environments [25,68,69], (ii) production of cytokines that regulate phagocytic cytocidal pathways to resist toxic reactive oxygen and nitrogen species, (iii) induction of anti-apoptosis pathways [26], (iv) modulation of cytokine balance to suppress proinflammatory processes elimination of infected cells and promote antinflammatory responses to enhance bacteria survival in infected cells [70,71], and (v) resist the killing effect of antimicrobial agents such as hepcidins [70]. Most of the intracellular bacterial pathogens causing disease in fish belong to the facultative bacteria species as previously classified by different scientists [72,73,74].

### 2.1. Edwardsiella tarda

*Edwardsiella tarda* is a facultative intracellular pathogen first reported in Japanese eel (*Anguilla japonica*) in 1962 in Japan [75] and characterized by Ewing et al. in 1965 [76]. Apart from fish, it has been isolated from different host species including snakes, amphibians, and mammals [77]. It enters macrophages by the clathrin- and caveolin-mediated endocytosis in which intracellular bacteria trafficking involves endosomes and endolysosomes [78]. Ling et al. [27] showed the invasion and internalization of *E. tarda* in vivo and in vitro using green fluorescent protein (GFP), while Qin et al. [28] attained internalization of the bacteria within 2 h after exposure to multiplicity of infections (MOIs) 10:1 and 100:1 in RAW264.7 cells. Once inside, the bacteria survive phagocytic defenses and replicates extensively in the macrophage vacuolar like compartments. T3SS and T6SS genes are essential for resisting phagocytic killing and are genetic hallmarks for distinguishing virulence from avirulent strains for *E. tarda* [18,26,79,80,81]. In addition, T3SS is required for intracellular replication and escape from infected macrophages, while T6SS is essential for intramacrophage infection as shown that T6SS mutants lose their adherence and invasion properties into macrophages [82]. Okuda et al. [26] showed that anti-apoptosis induction in *E. tarda*-infected cells was mediated by T3SS by upregulating NF-κB target genes such as Bcl2a1a, Bcl2a1b, cIAP-2, and TRAF-1 to protect infected macrophages from programmed cell death. In addition, T3SS was shown to downregulate IL-1β and block caspase-1-mediated cell death to prevent pyroptosis of *E. tarda*-infected macrophages. Tan et al. [18] showed that mutations involving genes such as esaB, escC, eseE, eseG, orf13, orf26, orf29, and orf30 in the T3SS gene cluster led to the failure of *E. tarda* to replicate in J774 macrophages and HEp-2 cells. They also showed that the virulence of *E. tarda* was severely affected by mutations of these genes in zebrafish (*Danio rerio*) in which they demonstrated that escC, orf13, orf19, orf29, and orf20 genes were required for intracellular replication and virulence [82]. *Edwardsiella ictaluri* modulates fish macrophage vacuolar pH and uses different proteins such as EsrA, EsrB, and EsrC and T3SS for its survival and replication in phagosomes of which mutational changes on some of these genes leads to failure of the bacteria to replicate in macrophages [83,56].

Nakhro et al. [84] and Srinivasa et al. [85] showed that *E. tarda* induces macrophages to produce superoxide anion and nitric oxide in common carp (*Catla catla*) and blue gourami (*Trichopodus trichopterus*) macrophages. They also showed that there was significant alteration in superoxide anion production by infected phagocytes, indicating that the bacteria was able to avoid or resist reactive-oxygen-species-mediated killing by the phagocytes. *E.-tarda*-induces macrophages to produce enzymes such as superoxidase dimutase (SOD), peroxidase, and catalase that are able to detoxify different reactive oxygen species (ROSs) and thus counteracts phagocyte-mediated killing [68,86]. Transcriptome analysis has shown that the bacterium has a broad range of inherent genes that are able to protect its cell wall from ROS damage, which broadens its capacity to cope with oxidative stress in order to enhance its survival intracellularly [69]. Apart from phagocytic cells, *E. tarda* infects epithelium papillosum of carp (EPC) cells, indicating that it is internalized by both phagocytic and non-phagocytic cells.

### 2.2. Piscirickettsia salmonis

*Piscirickettsia salmonis* is a facultative intracellular bacterium that causes hematocrit reduction and enlarged kidneys and spleen, in which all lymphoid tissues exhibit extensive necrosis leading to high mortalities [87,88]. The bacteria localizes in membrane bound cytoplasmic vacuoles in cells of lymphoid tissues. It propagates in macrophages and monocytes without inducing cytopathic effects (CPEs) [21] in which clathrin and actin cytoskeleton are required for its internalization into macrophages vacuoles [89]. Rojas et al. [90] showed that *P. salmonis* has the capacity to infect, survive, and replicate in the interior vacuoles of macrophages without inducing a characteristic cytopathic effect (CPE) by evading the phagolysosome activity and preventing the induction of apoptosis. McCarthy et al. [91] showed that the bacteria remains partially enclosed in the vacuole membranes, and escape into the cytoplasm is used as a means to avoid phagolysosomal fusion. Several nonphagocytic fish cell lines are also permissive to *P. salmonis* propagation in vitro such as chinook salmon embryo (CHSE)-214 from Onchorhynchus *tshawytscha*, Bluegill fry (BF)-2 fish cells from *Lepomis macrochirus*, CHum salmon Heart-1 (CHH-1) from *Onchorhynchus keta*, Coho Salmon Embryo *(CSE)-119 cells from O. kisutch*, EPC from *Cyprinus carpio*, and rainbow trout gonad-2 (RTG-2) from *O. mykiss* [21,90].

Genes shown to support intracellular survival of *P. salmonis* include clpB and bipA, which are upregulated during replication [92]. Both T3SS and T6SS are present in the *P. salmonis* genome although no studies have been done to demonstrate their functional mechanism in infected cells. Gomez et al. [93] showed that temporal acidification of cell free media resulted in overexpression of *P. salmonis* genes that inhibited phagosome-lysozyme fusion in order to prevent phagolysosome killing. To enhance its survival in infected cells, it upregulates antinflammatory cytokines such as IL-10 at early stages of its internalization to suppress induction of apoptosis and downregulates the expression of antimicrobial peptides (AMPs) such as hepcidin [70], demonstrating that it creates a cytokine imbalance that promotes its survival and replication in macrophages.

### 2.3. Yersinia ruckeri

*Yersinia ruckeri* is a facultative intracellular bacterium first reported in the Hagerman Valley, Idaho, USA in the 1950 as the causative agent of enteric red mouth disease (ERM) in fish. Since then, it has been reported to infect several fish species. Its adherence, invasion, and intracellular replication has been demonstrated ex vivo in different cell lines [14,15]. Ryckaert et al. [94] showed its replication in rainbow trout macrophages in which it produces reactive oxygen species (ROS) reaching a peak within a few hours after exposure. *Y. ruckeri* was able to survive and increase its replicate in toxic ROS microenvironments of macrophage vacuoles. Using electron microscopy they showed that bacteria were sequestered in autophagocytic compartments without fusion with primary lysosomes. Pujo and Bliska [95] pointed out that different Yersinia species rely on a common set of “core” virulence determinants to infect their host cells, which is in line with Tsukamo et al. [96] who pointed out that *Y. peudotuberculosis, Y. pestis*, and *Y. enterocolitica* share the ability to replicate and survive in macrophages by inhibiting phagosome acidification. These bacteria prevent phagosome maturation and production of nitric acid, which is also essential for killing of intracellular pathogens [96,97]. Afonso et al. [98] showed localization of *Y. ruckeri* in neutrophils that showed fusion of cytoplasmic granules with the phagosome using electron microscope. Immersion infection of juvenile rainbow trout resulted in a steady increase of bacteria replication in the headkidney extracellularly. As infection progressed, it resulted in a predominant intracellular replication phase in macrophages similar to observations made by other scientists [95] who showed that the majority of *Yersinia* species replicate extracellularly before gaining access into intracellular niches. In line with other intracellular bacteria species, various secretion system genes such as T1SS, T2SS, T3SS, and T4SS have been characterized from the *Y. ruckeri* SC09 genome [19], which could play a vital role in niche adaptation and pathogenesis of the bacteria. T3SS, commonly referred to as Ysa in Yersinia, shares the same chromosome encoded for *Y. enterocolitica* biotype 1B, suggesting that these bacteria mediate their biological functions by adapting to similar intracellular niches in infected cells [19].

### 2.4. Francisella noatunensis noatunensis and Francisella noatunensis orientalis

*Francisella noatunensis* is a facultative intracellular bacterium that produces granuloma lesions in various fish species such as Nile tilapia (*Oreochromis niloticus*), Atlantic salmon (*Salmo salar* L.), and Atlantic cod (*Gadus morhua*) [99,100,101]. *Francisella noatunensis noatunensis* survives and replicates in monocyte/macrophage cultures and epithelial like cells derived from Atlantic cod larvae cells (ACL cells), of which entry is mediated by phagocytosis [99]. Furevik et al. [100] used confocal microscopy to show the intracellular localization of *F. noatunensis* in Atlantic cod macrophages, monocytes, neutrophils, and B-cells. In early stages of infection, bacteria were observed more frequently in headkidney tissues than in peripheral blood and spleen leucocytes. In infected fish, bacteria were initially grouped close together near the nucleus and later were found in the cytoplasm suggesting that this could have been due to regression from the phagosome to the cytoplasm. Similarly, Bokkemo et al. [99] used transmission electron microscopy to show that the bacteria were enclosed in a phagosomal membrane during the initial phase of infection. At a later stage, bacteria were found in large electron-lucent zones surrounded by intact or disintegrated membranes. Immune electron microscope analysis showed the release of bacteria from intracellular vesicles pointing to phagosomal membrane disintegration allowing the bacteria to escape into the cytoplasm. Infected macrophages suppressed IL-1β and IL-8 proinflammatory cytokine expression, but upregulated IL-10 and IL-12/IL-17 antinflammatory cytokines. Vestivik et al. [71] showed that *F. noatunesis* inhibits respiratory burst in Atlantic cod leucocytes. Recently, T3SS was been detected in the *F. noatunensis orientalis* genome [59], which could play an important role in internalization, survival, and replication of the bacteria intracellularly as seen with other intracellular bacteria.

### 2.5. *Renibacterium salmoninarum*

*Renibacterium salmoninarum* is a facultative intracellular pathogen first reported in wild Atlantic salmon (*Salmo salar* L), brook (*Salvelinus fontinalis*), and brown trout (*Salmo trutta*) in the 1930s [46,47,48]. It is in the etiological agent for bacterial kidney disease (BKD), a chronic progressive granulomatous infection of salmonids mainly affecting the liver, kidney, and spleen. It survives and replicates in mononuclear phagocytic cells that protect bacteria from extracellular host defense mechanisms such as antibody binding and complement fixation [102]. In macrophages phagocytosis induces ROS and iNOS that does not inhibit replication of the bacteria [103]. The disease causes extensive tissue damage, induces a strong CMI response, macrophage proliferation, and activation, as well as deposition of immune complexes and type III hypersensitivity reaction [104]. The bacteria modulates host immune response to its advantage by interfering with cytokine responses and suppressing production of oxygen species (ROS) and antibody responses [105,106]. It uses its endogenous proteins such as p57 to suppress the expression of proinflammatory cytokines such as IL-1β, which also induces a chronic reduction in MHC-II expression and skews the T-cell responses toward the MHC-I pathway.

### 2.6. Other Intracellular Replicating Bacteria

*Vibrio parahaemolyticus* uses macrophages and neutrophils as its primary replication sites in infected fish, and its intracellular replication has been shown in *Epinephelus awoara* phagocytic cells [107]. Deletion of T6SS in *V. parahaemolyticus* reduces bacteria adhesion to monocytes and render the bacterium avirulent because of its inability to gain intracellular replication capacity [108]. The pathogens associated with epitheliocystis are intracellular replicating pathogens reported in Atlantic salmon, leafy seadragon (*Phycodurus eques*), silver perch (*Bidyanus bidyanus*), and barramundi (*Lates calcarifer*) that include *Candidatus* Piscichlamydia salmonis taxonomically allocated within the order Chlamydiales [49,109]. *Mycrobacteria marinum* is an intracellular fish pathogen that replicates in macrophages [110]. Intravascular mycobacterium infections block phagosome-lysosome fusion, so vacuoles containing the bacteria do not acidify to pH < 6.5 as a survival strategy by blocking establishment of acidic environments in the vacuoles where they replicate. Several *Mycobacterium* spp. have been reported in fish associated with chronic granulomatous lesions (Table 1). Unlike other intracellular bacteria that use T3SS and T6SS genes for their survival, *Mycobacterium* spp. use T7SS for intracellular trafficking of proteins that enhance their survival in macrophages [111,64] (Table 2), which has also been detected in the *M. marinum* genome. Other intracellular bacteria include *Photobacterium damselae* subsp. *piscicida* the causative agent of photobacteriosis formerly known as fish pasteurellosis or pseudotuberculosis, which produces superoxidase dismutase (SOD) and catalase enzymes when exposed to oxidative stress as a survival strategy in macrophages [65]. Barnes et al. [65] showed that virulent strains with higher capacity to produce SOD and catalase had high survival and replication capacity in sole (*Solea senegalensis*, Kaup) phagocytes than the avirulent strains.

## 3. Vaccination and Adaptive Immune Responses

The central hallmark of vaccination is to prime the adaptive immune system by exposure to antigens of pathogens so that in subsequent exposure the immune system will mount a rapid protective response against the same pathogen. Like all vertebrates, the adaptive immune system of teleosts fish is subdivided into (i) cellular mediated immunity, whose mode of protection is to “kill” and eliminate pathogen-infected cells, and (ii) humoral immunity that depend on antibodies to neutralize pathogens in body fluids.

### 3.1. Cellular Mediated Immunity

Bacteria that replicate inside host cells are inaccessible for antibody neutralization in the extracellular matrix. The most effective adaptive immune protective mechanism against such pathogens is to “kill” and eliminate infected cells by the cellular mediated immune system. To do this, CD8 T-cells recognize infected cells by binding to MHC-I molecules expressing peptides processed from intracellular pathogens. The MHC-I ligands bind to their respective T-cell receptors (TCR) on the surface of CD8+ T-cells. It is noteworthy that all TCRs (α, β, γ, and δ) found in mammalian CD8 T-cells have been characterized in fish CD8 cells [112,113,114]. In addition, CD28 costimulatory and CTLA-4 negative regulatory markers that mediate the interaction between CD8+ cells and MHC-molecules have also been characterized in fish [115,116]. Upon binding to MHC-I molecules, naïve CD8 cells are activated into effector cytotoxic T-lymphocytes (CTLs) that secrete cytotoxic granules containing perforins and granzymes. Perforins form spores in target cell membranes enabling granzymes, which are serine protease enzymes, to enter the target cells and cleave to host proteins in order to induce apoptosis. To execute their effector functions, CD8+ cells are helped by CD4+ cells. Characteristic cytokine signatures mediate the differentiation of naïve CD4+ cells into different effector helper (Th) subtypes. For example, the differentiation of naïve CD4+ cells into Th1 cells is mediated by cytokines such as TNFα, IFNγ, and TGFβ, while specification into Th2 cells is mediated by cytokines such as IL-4/13 and IL-6. These cytokines play crucial roles in a paracrine and autocrine modulation of macrophage in activation of CD8+ cells against intracellular pathogens as well as induction of humoral immune responses against extracellular pathogens. Hence, a good understanding of cytokine signatures that skew CD4+ cells toward Th1 differentiation could serve as immune-adjuvants able to prime CD8+ cells in producing protective CMI responses against intracellular pathogens [7,117,118].

#### 3.1.1. *Edwardsiella tarda* Cell-Mediated Immunity

Suffice to mention that the CMI responses against *E. tarda* have been studied in more detail using ginburna carp (*Carassius auratus langsdorfii*) than other intracellular bacteria species in fish [119]. Yamasaki et al. [120] showed higher protection in ginburna carp vaccinated against *E. tarda* using a live vaccine than with a WCI vaccine. The live vaccine induced high CD4+ and CD8+ T-cell responses alongside high antibody responses compared to the WCI vaccine that only induced antibody responses when CD4 and CD8 responses were suppressed. These findings show that vaccines that induce both high CMI and antibody responses produce higher protection than vaccines that only produce antibody responses. In addition, they observed an upregulation of IFNγ and T-bet that corresponded with an increase in CTL responses. To consolidate the findings that suggest that the CMI response is crucial for protection against Edwardsiellosis, Yamasaki et al. [121] adoptively transferred T-cells sensitized by *E. tarda* into isogenic naïve ginbuna crucian carp and showed that recipients of CD4+ and CD8+ cells acquired significantly high protection against *E. tarda* after challenge. They further observed that the passive transfer of CD8+ cells upregulated IFNγ and perforin consolidating the notion that protective immunity against *E. tarda* was highly dependent on IFNγ-mediated cell cytotoxicity. Matsuura et al. [122] showed enhanced expression of granzyme in ginburna crucian carp exposed to *E. tarda* infection by allosensitization. In addition, the direct antibacterial killing activity of *E. tarda* by activated CD8+ cells has been demonstrated by Nayak and Nakanishi [123,124]. Nayak and Teruyuki [123] further showed that CD4+ and CD8+ T-cells sensitized by *E. tarda* vaccination had a higher antibacterial activity than non-sensitized cells, demonstrating that immunization increases CTL effector functions. Moreover, the costimulatory CD28 molecule was upregulated in lymphoid organs of fish infected by *E. tarda*, indicating that this gene could play a vital role in activating CD8+ responses in fish [125].

#### 3.1.2. *Edwardsiella tarda* Immunogenic Proteins for Cellular Mediated Immune Responses

Fang et al. [126] showed that *E. tarda* proteins such as EscE (Orf13) are involved in the activation of CD4+ and CD8+ T-cell responses in fish. Mahendran et al. [127] used a computer-aided vaccine design approach for the prediction of proteins from the *E. tarda* outer membrane protein (Omp) that interact with MHC-I alleles. Two epitopes from *E. tarda* Omp exhibited excellent protein–peptide interaction when docked with MHC-I class alleles. Sun et al. [128,129] compared the efficacy of a subunit (rEta2) and DNA (pCEta2) vaccine made from Eta2 protein spanning 178 residues. The DNA vaccine (pCEta2) upregulated IFNγ, Mx, CD8α, MHC-1α, and IgM, while the subunit vaccine (rEta2) only upregulated IL-1β, complement C3, and IgM. Taken together, their findings showed that the DNA vaccine (pCEta2) induced both B- and T-cell responses, whereas the subunit vaccine (rEta2) only induced humoral responses. In another study, Sun et al. [129] cloned the Esa1 protein and produced a DNA vaccine (pCEsa1) that produced high protection accompanied by increased respiratory burst activity, bactericidal activity in headkidney macrophages, serum bactericidal activity in a ca(2+)-dependent manner, high IgM levels and upregulation of Th1 cytokine genes. Comparative analyses showed that the subunit vaccine (rEsa1), made from the gene (Esa1), was less protective than the DNA (pCEsa1) vaccine under severe challenge resulting in 92–97% mortality consolidating observations that replicative vaccines are more protective than non-replicative vaccines [130,131]. Similarly, Yang et al. [132,133] used RNA-seq to show that a live *E. tarda* vaccine revealed an activated MHC-I pathway and inhibited the MHC-II pathway during the early stages of response to immunization in zebrafish. They showed upregulation of the MHC-I pathway and activation of the CTL response when MCH-II pathway was downregulated. Other immunogenic proteins having the potential to serve as vaccine candidates for CMI responses against *E. tarda* are shown in Table 3.

#### 3.1.3. Cellular Mediated Immunity Induced by Other Intracellular Bacteria

Comparison of fish intracellular bacteria species with their mammalian counterparts suggests that fish pathogens could be using mechanisms similar to mammalian pathogens in evoking CTL responses. Nagata and Koide [10] have pointed out that Yersinia infected vacuoles in macrophages are acidified by fusion with lysosomes leading to induction of CD4+ and CD8+ responses. Given that most Yersnia species use similar “core” survival and replication strategies in phagocytic cells, as pointed out by Puyol and Bliska [95], it is likely that similar CD4+ and CD8+ induction mechanisms might be in existence for *Y. ruckeri* in fish cells. A live vaccine against *P. salmonis* led to a significant reduction in mortality [142] compared to a WCI vaccine, while a transcriptome-based study done by Rozas-serri et al. [143] showed that *P. salmonis* skewed cytokine production toward IFNγ resulting in Th1 polarization and induction of CD8+ and CD4+ T-cell responses. Similarly, Bakkemo et al. [99] showed increased expression of IL-12/IL-17 that was linked to polarization of Th1 responses in Atlantic cod macrophages exposed to *P. salmonis*. In the case of *R. salmoninarum*, it has been shown that it uses its endogenous proteins such as p57 to suppress the expression of proinflammatory cytokines, such as IL-1β and MHC-II response. Consequently, it skews the T-cell responses toward MHC-I and CMI responses. In summary, these studies show that CMI-based vaccines are protective against intracellular bacterial infection than the non-replicative vaccines. Immunogenic proteins linked to the induction of CMI responses in *P. salmonis* and other bacteria species, excluding E. tarda, are shown in Table 4 and Table 5, respectively. In general, these studies show that more efforts have been directed at producing replicative vaccines using live attenuation and recombinant DNA technologies to generate CMI-based vaccines.

### 3.2. Humoral Immune Responses

There is a long successful history of vaccination against bacterial fish diseases mainly attributed to empirical vaccines targeting extracellular pathogens [159]. Conversely, the design of protective vaccines against intracellular bacterial infections is a challenge. This is because intracellular pathogens evade antibody neutralization by growing inside the host cells in which antibody responses induced by extracellular vaccines cannot neutralize the pathogens [160]. Park et al. [161] noted that much as progress in the search of protective vaccines against *E. tarda* has led to discovery of numerous vaccine candidates, these efforts have not been translated into commercial vaccines. Yamasaki et al. [162] observed that antibody responses from live vaccines only increased after bacteria clearance by CTLs, indicating humoral immune responses appears too late to provide protective immunity in fish vaccinated against *E. tarda*. Similarly, Evelyn et al. [106] showed that antibodies for *R. salmoninarum* were not protective against post-challenge infection, which is in line with observation made for *P. salmonis* that the majority of WCI vaccines fail to produce protection in vaccinated fish. Similarly, Cossarini-Dunier [163] suggested that protection against *Y. ruckeri* did not seem to be dependent on antibodies. Further, Raida and Buchmann [164] showed that passive immunization using transfer of plasma from vaccinated fish to naïve fish conferred no protection, suggesting that humoral responses such as IgM and complement are less protective against *Y. ruckeri*. The lack of in-vitro culture methods for Chlamydial pathogens makes it difficult to develop vaccines against epitheliocystis, so immune protective mechanisms based on vaccination have not been reported [165]. Overall, these studies show that humoral immune responses are not protective against intracellular pathogens in fish. However, in some cases, antibodies produce protection possibly by reacting with pathogens shortly after infection before entry into host cells or during cell-to-cell transmission. For example, *Y. ruckeri* does not survive intracellularly for a long time [94,166,167] and thus has an extracellular replication phase before entry into the intracellular compartments [95,98]. It is likely that antibodies neutralize the bacteria during extracellular replication before entry into the intracellular niches [168], which is contrary to observation made by Cossarini-Dunier [163] and Raida and Buchmann [164]. However, there is a need for detailed investigation aimed at identifying the most appropriate timing when vaccination would block infection from progressing into the intracellular phase by neutralizing the pathogens during the extracellular phase. Overall, the general observation shows that humoral immune responses are not protective against intracellular bacterial infections. Hence, the challenge is to develop protective CMI based vaccines.

## 4. General Discussion and Conclusions

One of the outstanding challenges of vaccine design for intracellular bacteria diseases is that the mechanisms of CMI response have not been elucidated for most fish species. The main driving force for vaccine research in aquaculture is the commercialization of fish production. Hence, fish species such as Nile tilapia (*Oreochromis niloticus*) that have recently gained commercial importance to become the second farmed species in the world in the last five years [169] lag behind in terms of immunology and vaccinology research than Atlantic salmon and rainbow trout (*Oncorhynchus mykiss*) that have been under commercial production for a long time. Therefore, several CMI studies have been carried out in salmonids than Nile tilapia. For example, the CD8α and CD8β genes characterized as molecular markers of activated CD8+ cells in salmonids [170] have not been characterized in Nile tilapia. TCRs chains together with their corresponding ligands on MHC molecules characterized in salmonids [171] have not been characterized in Nile tilapia. Previously, we used gene expression to elucidate the kinetics of CD4+ and CD8+ cells alongside their transcription factors such as GATA-3, T-bet, and eomesdermin in Atlantic salmon vaccinated against infectious pancreatic necrosis virus (IPNV) [172] of which most of these transcription factors have not been characterized in tilapia. There is need for the characterization of adaptive immunity genes for fish species entering commercial production in order to expedite the process of developing protective vaccines for both extra- and intracellular pathogens.

To prime the CMI with the protective capacity against subsequent infection, it is important that intracellular antigens activate the CD8+ T-cells. However, factors limiting the design of highly protective vaccines against intracellular fish pathogens are still a challenge. Cellular mechanisms leading to activation of CD8+ cells by various bacterial species localizing different intracellular niches have not been clearly elucidated in fishfish as done for viral pathogens [172,173,174]. Moreover, the network pathways evoked by intracellular bacteria have not been studied in detail as done in the case of viral infections [174,175,176]. The duration for which CD8+ cells activated by vaccination remain effective in “killing” infected cells has not been determined. As a result, optimal levels of CD8+ cell activation by vaccination able to confer protective immunity have not been determined. Thus, the measures of efficacy for activated CD8+ cells as a protective endpoint that correlate with post challenge survival proportions (PCSPs) in vaccinated fish are unknown. This is contrary to studies on humoral immune responses in which antibody titers that serve as protective endpoint correlating with PCSP in vaccinated fish have been determined for different vaccines [119,177,178,179,180,181]. Moreover, it remains unknown the extent to which mucosal vaccination can evoke protective immunity against intracellular replicating bacteria unlike in the case of viral and extracellular bacterial infections in which protective mechanisms have been widely studied [182,183,184,185]. Further, concurrent involvement of Th1 and Th2 responses in conjuring protective immunity is also a challenge given that optimal vaccine conditions that produce CD4+ levels able to potentiate CD8+ cells to produce protective immunity in vaccinated fish have not been determined. Hence, the practical conditions in which intracellular vaccination translates into protective CMI responses have not been determined for most fish vaccines. Until all these parameters are studied in detail as an overture to findings solutions to immunological pitfalls limiting the design of protective vaccines, production of highly protective vaccines for intracellular bacteria remains a challenge. Thus, the ultimate challenge is to apply our limited understanding of CMI responses in designing protective vaccines for intracellular bacteria in fish. Existing evidence shows that the production of CMI-based vaccines would be the most effective approach able to reduce persistence of outbreaks caused by intracellular bacterial infections adversely affecting the aquaculture industry.

## Figures and Tables

**Table 1:** Intracellular bacteria species characterized in fish.

Bacteria	Host species	References
*Piscirikettsia salmonis*	Salmonids	[21,22,23,24]
*Edwardsiella tarda*	Various species	[25,26,27,28,29,30]
*Edwardsiella ictaruli*	Various species	[31]
*Yersinia ruckeri*	Various species	[14,15]
*Francisella noatunensis* subsp. *Orientalis*	Chichilds and other warm water species	[32,33]
*Francisella noatunensis* subsp. *noatunensis*	Atlantic cod (*Gadus morhua*) Atlantic salmon (*Salmo salar L*)	[34,35,36]
*Vibrio parahaemolyticus*	Various species	[37]
*Photobacterium damselae* subsp. *piscicida*	Various species	[38,39]
*Candidatus* piscichlamydia salmonis	Salmonids	[40,41,42]
*Mycobacterium marinum*	Various species	[43,44]
*Mycobacterium chelonae*	salmonids	[45]
*Mycobacterium gordonae*	Various species	[43]
*Mycobacterium fortuitum*	Various species	[43]
*Mycobacterium trivale*	Various species	[43]
*Renibacterium salmoninarum*	Various species	[46,47,48]
*Candidatus pisciclamydia salmonis*	Salmonids	[49]
Tasmanian Rickettsia-like organism (RLO)	Salmonids	[50,51]

**Table 2:** Secretion system proteins for intracellular bacteria trafficking fish pathogens.

Bacteria species	Protein	Abbr	Reference
*Edwardsiella tarda*	Type III secretion system	T3SS	[52,53]
*Edwardsiella tarda*	Type VI secretion system	T6SS	[53]
*Piscirikettsia salmonis*	Type III secretion system	T3SS	[54,55]
*Piscirikettsia salmonis*	Type VI secretion system	T6SS	[54,55]
*Edwardsiella ictaruli*	Type III secretion system	T3SS	[56]
*Francisella* spp.	Type IV secretion system	T4SS	[33,57,58]
*Franciesella natunensis*	Type IV secretion system	T4SS	[59]
*Vibrio parahaemolyticus*	Type IV secretion system	T3SS	[60,61]
*Vibrio parahaemolyticus*	Type IV secretion system	T6SS	[61]
*Yersinia ruckeri*	Type I secretion system	T1SS	[62]
*Yersinia ruckeri*	Type II secretion system	T2SS	[19]
*Yersinia ruckeri*	Type III secretion system	T3SS	[19]
*Yersinia ruckeri*	Type IV secretion system	T4SS	[19]
*Edwardsiella piscicida*	Type VI secretion system	T6SS	[63]
*Mycobacterium* spp.	Type VII secretion system (ESX1–5)	T7SS	[64,65]
*Photobacterium damselae* subsp. *piscicida*	Type II secretion system	T7SS	[66]
*Candidatus* Ichthyocystis sparus	Type II secretion system	T2SS	[42,67]
*Candidatus* Ichthyocystis sparus	Type III secretion system	T3SS	[42,67]

**Table 3:** *Edwardseilla tarda* immunogenic proteins used in recombinant vaccine production.

Protein	Fish species	Mode	Ref.
Outer membrane protein C	Japanese flounder (*Paralichthys olivaceus*)	Subunit	[134]
Outer membrane protein (Omp85)	Rohu (*Labeo rohita*)	Subunit	[135]
Outer membrane protein (Omp45)	Rohu (*Labeo rohita*)	Subunit	[136]
Outer membrane protein A	Japanese flounder (*Paralichthys olivaceus*)	Subunit	[137]
Outer membrane protein ToIC	Japanese flounder(*Paralichthys olivaceus*)	Subunit	[127]
Eta2	Japanese flounder (*Paralichthys olivaceus*)	Subunit	[128]
Eta2	Japanese flounder (*Paralichthys olivaceus*)	DNA	[128]
Esa1	Japanese flounder (*Paralichthys olivaceus*)	DNA	[129,130]
Esa1	Japanese flounder (*Paralichthys olivaceus*)	Subunit	[129,130]
Eta21	Japanese flounder (*Paralichthys olivaceus*)	Subunit	[130]
DH5alpha/pTAET21	Japanese flounder (*Paralichthys olivaceus*)	live	[130]
pCE18	Japanese flounder (*Paralichthys olivaceus*)	DNA	[138]
pEta6	Japanese flounder (*Paralichthys olivaceus*)	DNA	[138]
pCE6	Japanese flounder (*Paralichthys olivaceus*)	DNA	[138]
pCE18	Japanese flounder (*Paralichthys olivaceus*)	DNA	[138]
Outer membrane protein A	Rohu (*Labeo rohita*)	Subunit	[139]
DegP	Japanese flounder (*Paralichthys olivaceus*)		[140]
OmpA	Rohu (*Labeo rohita*)	Subunit	[141]

**Table 4:** *Piscirikettsia salmonis* immunogenic proteins used in recombinant vaccine production.

Protein	Fish species	Ref.
Membrane vesicles	Zebrafish (*Danio rerio*)	[144]
Heat shock proteins Hsp60 and Hsp70	Atlantic salmon (*Salmo salar* L.)	[145]
Heat shock proteins Hsp10 and Hsp16	Atlantic salmon (*Salmo salar* L.)	[146]
Membrane bound transglycosylase B (MltB)	Atlantic salmon (*Salmo salar* L.)	[147]
Transferring binding protein B (TbpB)	Atlantic salmon (*Salmo salar* L.)	[147]
ChaPs	Atlantic salmon (*Salmo salar* L.)	[148]
Outer surface lipoprotein A (OspA)	Atlantic salmon (*Salmo salar* L.)	[149,150]
Slt70	Atlantic salmon (*Salmo salar* L.)	[146]
Omp27	Atlantic salmon (*Salmo salar* L.)	[146]
FlgF	Atlantic salmon (*Salmo salar* L.)	[146]
FlgG	Atlantic salmon (*Salmo salar* L.)	[146]
FlgH	Atlantic salmon (*Salmo salar* L.)	[146]
FlaA	Atlantic salmon (*Salmo salar* L.)	[146]

**Table 5:** Immunogenic proteins used for recombinant vaccine production for other bacteria species.

Bacteria species	Fish species	Type	Ref.
*Yersinia ruckeri* iron regulated Omp	Rainbow trout (*Oncorhynchus mykiss*)	Subunit	[151]
*Yersinia ruckeri* Serralysin metalloprtease (Yrp1)	Rainbow trout (*Oncorhynchus mykiss*)	Subunit (toxoid)	[152]
Outer membrane vesicles	Zebrafish (*Danio rerio*)	vesicles	[153,154]
*Vibrio parahaemolyticus* OmpK	Seabream (*Acanthopagrus schlegelii*)	DNA	[155]
*Vibrio parahaemolyticus* OmpV	yellow croaker (*Pseudosciaena crocea*)	Subunit	[156]
*Vibrio parahaemolyticus* OmpU	yellow croaker (*Pseudosciaena crocea*)	Subunit	[156]
*Vibrio parahaemolyticus* OmpW	yellow croaker (*Pseudosciaena crocea*)	Subunit	[156]
*Vibrio parahaemolyticus* TolC	yellow croaker (*Pseudosciaena crocea*)	Subunit	[156]
*Vibrio parahaemolyticus* Serine protease gene	turbot (*Scophthalmus maximus*)	DNA	[157]
*Mycobacterium marinum* Ag85 gene	Hybrid striped bass (*Morone saxatilis* x *M. chrysops*)	DNA	[158]
